# Effects of Ageing, Oestrogen Level and Altered Dietary Loading on Rat Mandibular Cartilage—A Polarised Light Microscopy Study

**DOI:** 10.1111/ocr.70010

**Published:** 2025-08-09

**Authors:** Riikka Hauru, Bijay Shakya, Lassi Rieppo, Anna‐Sofia Silvola, Jia Yu, Sakari Laaksonen, Simo Saarakkala, Aune Raustia, Pertti Pirttiniemi

**Affiliations:** ^1^ Research Unit of Population Health, Faculty of Medicine University of Oulu Oulu Finland; ^2^ Medical Research Center Oulu University Hospital and University of Oulu Oulu Finland; ^3^ Research Unit of Medical Imaging, Physics and Technology University of Oulu Oulu Finland; ^4^ The Fourth Military Medical University Xi'an China; ^5^ Laboratory Animal Centre University of Oulu Oulu Finland

**Keywords:** condylar cartilage, PLM, rat, TMJ

## Abstract

**Objective:**

The mandibular condylar cartilage (MCC) of rats was examined with polarised light microscopy (PLM) to study the effects of ageing, oestrogen level, and altered dietary loading on the structure of the MCC.

**Materials and Methods:**

96 Sprague–Dawley rats were separated into 12 groups based on their age (5 months [young] and 14 months [old]), oestrogen status (ovariectomised [OVX], non‐ovariectomised [non‐OVX]), and diet (hard, normal, or soft). The MCC specimens were examined using PLM to evaluate the orientation and retardation of collagen fibrils. The MCC was segmented sagittally into three distinct regions: anterior, most superior, and posterior. For each segment, the PLM values at varying depths were statistically compared across different groups using an N‐way analysis of variance (ANOVA).

**Results:**

Ageing significantly increased collagen fibril orientation angles with respect to cartilage surface across all segments, particularly in the superficial layer, indicating structural modifications. Oestrogen deficiency (OVX) tended to increase fibril orientation angles and caused incoherence in retardance. In the anterior segment, a harder diet increased fibre orientation angles, while in the most superior segment it decreased retardance, further impacting collagen structure.

**Conclusion:**

Age and dietary loading significantly influenced collagen fibril orientation and retardance, with ageing generally leading to increased fibril orientation angles and often resulting in increased retardance. Dietary variations, particularly harder diets, emerged as a key factor with more substantial effects on collagen structure, affecting both fibre orientation and retardance.

## 1 Introduction

The temporomandibular joint (TMJ) is a heavily loaded joint and has a fibrocartilage layer in the mandibular condylar head. It also has unique healing and adaptive properties in adverse environmental conditions [1]. The mandibular condylar cartilage (MCC) is primarily composed of collagen fibrils and proteoglycans, providing a viscoelastic response to loading and acting as a stress absorber during mastication [2, 3]. Type I, Type II, and Type X collagens are expressed in the MCC [4]. Type I collagen is expressed in the superficial or fibrous layer [5, 6]. Type II collagen, being the most abundant, is expressed in mature and upper hypertrophic layers [5, 6], and Type X collagen is expressed in the mature matrix in the thin layer of calcified cartilage [4]. Collagen fibril orientation angles, the amount of cartilage cells, and matrix composition vary at different depths of cartilage [7].

As fibrocartilage ages, proteolytic activity increases, and the secretion of proteoglycans decreases, which finally leads to cartilage degradation [8, 9]. These age‐related changes also compromise the structural and functional integrity of the MCC, making it more vulnerable to degenerative conditions. Oestrogen plays a pivotal role in maintaining the structural integrity and functionality of the TMJ, underscoring its influence on cartilage remodelling and bone density [1, 10, 11]. Hormonal fluctuations, particularly a reduction in oestrogen, have been shown to accelerate cartilage degradation and reduce collagen content.

Biomechanical loading influences mandibular condylar cartilage growth by modulating extracellular matrix composition and collagen fibre alignment, highlighting the interaction between mechanical and hormonal factors [2, 12]. To maintain optimal chondrocyte proliferation and matrix production, proper loading is an important factor in mandibular cartilage growth [12, 13]. Changes in mechanical loading patterns, such as reduced masticatory activity, can alter cartilage remodelling and reduce the tissue's capacity to adapt to physiological stress. Hadaegh et al. [14] demonstrated that low‐intensity pulsed ultrasound (LIPUS) stimulates both chondrogenesis and osteogenesis within the mandibular condyle of young adult rats, increasing chondroblast density and fibrocartilage thickness. Histomorphometric evaluations by Hadaegh and El‐Bialy [15] further showed that LIPUS significantly increased fibrocartilage thickness and cell density, particularly in the middle region of the condyle.

Articular cartilage is a highly specialised tissue that lines the heads of bones in joints, providing a low‐friction surface for smooth movement [16]. Collagen orientation in articular cartilage is critical for the biomechanics and pathology of the tissue [17]. Different regions of articular cartilage have distinct collagen orientation patterns that affect its mechanical properties and resistance to wear and tear [18, 19].

The collagen network architecture of articular cartilage can be visualised using polarised light microscopy (PLM). In addition to visualisation, it is also possible to quantify the orientation and retardation of collagen fibrils with PLM [20]. This is advantageous for more detailed analysis of collagen network properties. In PLM, the sample is illuminated with polarised light, and in birefringent samples, polarised light interacts with the sample, generating contrast against the background of the samples [20].

Retardation in PLM refers to the phase shift experienced by polarised light as it passes through a birefringent material, such as collagen fibrils in cartilage [9, 20]. This phase shift occurs because the speed of light through the material varies along different axes due to the anisotropic structure of the collagen network. The degree of retardation is influenced by the thickness, density, and orientation of the collagen fibrils [20]. In studies by Mirahmadi et al. [9], retardation measurements provided insights into the biomechanical properties and changes in the collagen matrix.

Orientation refers to the alignment or direction of collagen fibrils within the cartilage matrix with respect to the cartilage surface and is determined by analysing the angle of polarised light transmitted through the sample to reveal the predominant direction of the collagen fibrils. Changes in the orientation of these fibrils can indicate alterations in the structural integrity and functional properties of the cartilage. Studies such as those by Kuroda et al. [2] and Delatte et al. [6] have explored the biomechanical and biochemical characteristics of cartilage, including the orientation of collagen fibrils, to understand their role in joint function and responses to physiological or pathological conditions.

The collagen network architecture of MCC in mice and rabbits has been analysed by Hunziker et al. [19] and Vanden Berg‐Foels et al. [21], employing techniques such as polarised light microscopy, scanning electron microscopy, and helium ion microscopy. Ojanen et al. [22] utilised PLM to validate collagen fibril orientations obtained from micro‐computed tomography (μCT) imaging. PLM images were used to map collagen orientation across tissue depth in histological samples, with pixel values representing the orientation angle relative to the cartilage surface. This analysis across depth allowed for a comparison between the orientation profiles obtained from μCT imaging and those from PLM, demonstrating a strong correlation between the two methods despite some systematic differences in the values obtained [22].

The aim of this study was to examine the effects of ageing, oestrogen level, and altered dietary loading on collagen fibril orientation angles and organisation in rat MCC by using PLM. Another aim was to find the precise location of changes in the MCC of rats. The hypothesis was that ageing, oestrogen level, and altered dietary loading have a significant effect on the collagen fibril orientation angles, retardation, and parallelism in rat MCC.

## 2 Materials and Methods

### 2.1 Material

The study included a total of 96 outbred female Sprague–Dawley rats that were further divided into 12 distinct subgroups based on specific criteria [11, 23]. These criteria included age, with rats categorised as either “young” (5 months) or “old” (14 months); oestrogen status, dividing rats into either “ovariectomised” [OVX] or “non‐ovariectomised” [non‐OVX]; and diet type, with three dietary categories encompassing “hard” [diet board], “normal” [pellet], and “soft” [powder], as outlined in Table S1. To delve into the influence of oestrogen deficiency on the MCC, half of the rats were ovariectomised at the age of 7 weeks. The sample material has been described in detail previously [11]. The diet board feeding method imposed a higher workload on the condylar cartilage compared to a normal diet, requiring rats to engage in wood gnawing behaviour to access their food sources [10].

The rats underwent pre‐anaesthesia with isoflurane and at the conclusion of the experiment, they were humanely euthanised using 100% carbon dioxide. To ensure the preservation of the craniums, they were immersed in a 4% formalin solution for 7 days, followed by decalcification using EDTA (pH 7.4) at 37°C for 6 weeks. After this decalcification process, the craniums were sagittally hemi‐sectioned, yielding two portions, each containing a left and right temporomandibular joint (TMJ). These sections were subsequently embedded in paraffin. For this study, we focused exclusively on the right TMJs, which were further processed by cutting into 5‐μm thick sagittal sections to facilitate subsequent analysis and staining procedures. Specifically, the most central sagittal sections of the condyle were selected for analysis and confirmed using toluidine blue staining. Details about the sample materials have been explained in a previous publication [11].

### 2.2 Methods

Samples were examined with PLM to provide insight into the alignment of Type I and Type II collagen networks in rats' condylar cartilage. Unstained 5‐μm‐thick MCC segments were studied with the Abrio PLM system (Cri Inc., Woburn, MA, USA), which was mounted on a conventional light microscope (Nikon Diaphot TMD, Nikon Inc., Shinagawa, Tokyo, Japan). The Abrio system is equipped with a green bandpass filter, a circular polariser, and a computer‐controlled analyser that utilises two liquid crystal polarisers coupled with a CCD camera for image acquisition. The Abrio PLM system measures the birefringence of tissue sections by detecting the retardance, or phase shift, caused by the interaction of polarised light with anisotropic structures in the sample. As polarised light passes through birefringent material, it is split into two orthogonal components, each travelling at different speeds, creating a phase difference. This system captures the magnitude of retardance, providing quantitative information on the optical anisotropy of the sample [24] (Figure S9).

**FIGURE 1:**
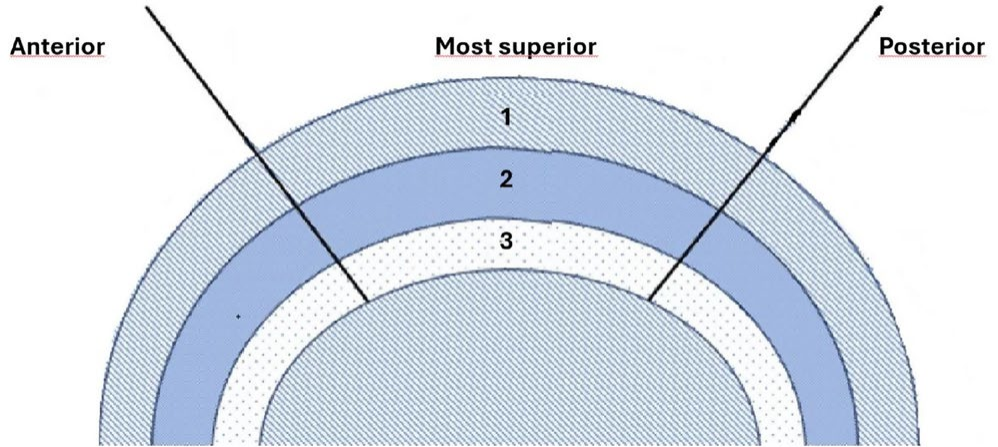
Schematic drawing of a rat condylar head divided sagittally into three segments (anterior, most superior, posterior); from each segment, three layers were taken from three depths. The layers were divided as follows: superficial layer (depth 1), middle layers (depth 2) and deep layer (depth 3).

After the acquisition of images, three segments were selected from the MCC and these were divided sagittally into anterior, most superior, and posterior regions. Each of these segments was further divided into a superficial layer (depth 1, 0%–18% of cartilage thickness from the cartilage surface), middle layer (depth 2, 18.8%–68.8%), and deep layer (depth 3, 68.8%–100%), as shown in Figure 1. Regions of interest (ROIs) were manually selected within each of the three segments of the images from the outer border of the condyle to the edge of the subchondral bone. When selecting the ROIs, the width remained constant for each segment across all samples, while the height was adjusted to match the cartilage thickness. The average depth‐dependent profile for each measurement location was then calculated.

The orientation profile contains information about the orientation of collagen fibrils in the MCC, while the retardation profile reflects the collagen content and uniformity of the fibre alignment. The depth‐dependent profiles were scaled to 100 points to enable direct comparison between the specimens. The analysis was performed using a custom algorithm in MATLAB (R2021a, MathWorks, Natick, MA, USA).

A statistical power analysis was performed to confirm that the sample size was adequate for detecting biologically relevant differences in mandibular condylar cartilage. Computer software (https://www.stat.ubc.ca/~rollin/stats/ssize/n2.html) was used to calculate the sample size to obtain valid results. The required effect size was obtained from measurements in a prior study conducted by Orajärvi et al. [25]. A power of 0.8 was chosen for the experiment and, to achieve this level of statistical power, a total of 8 animals was determined to be necessary for each group [26].

Collagen orientation was analysed using polarised light microscopy (PLM), a well‐established method for assessing birefringence and collagen fibril alignment in articular cartilage [20, 24]. The retardation profile, reflecting collagen density and uniformity, was quantified using a combination of high‐resolution imaging and computational analysis, following well‐documented protocols in the literature [22, 23], with the method described in more detail in the Appendix.

### 2.3 Statistical Analyses

In this study, we conducted a statistical comparison of the groups employing N‐way analysis of variance (ANOVA) to assess the significance of our findings. A *p*‐value of less than 0.05 was considered statistically significant. The N‐way ANOVA included ageing, oestrogen level, and altered dietary loading as the factors under investigation. We examined both the individual and combined effects of these factors across all cartilage depths. The initial phase of our analysis focused on evaluating how age, oestrogen level, and altered dietary loading influenced the orientation and retardation of the collagen network within the MCC. In the subsequent phase, we compared the various groups within three distinct layers. To facilitate this comparison, we calculated the average data points for each sample within these layers. P‐values were subjected to correction using Tukey's honest significant difference criterion to minimise false differences between groups. All statistical analyses were performed using MATLAB (R2021a, MathWorks, Natick, MA, USA).

## 3 Results

### 3.1 Age

The present findings showed a definitive age‐related transformation in the collagen framework of the MCC. Older rats consistently exhibited higher fibril orientation angles in the anterior and posterior segments' layers, which indicate age‐induced structural changes (*p* < 0.05) (Figures S5A,C, S7A,C). The trend extended to the most superior segment, where older rats displayed a similar pattern of higher angles, highlighting the influence of age on collagen orientation (Figures S2C, S7B). Retardance profiles revealed age‐related changes in collagen fibril orientation, with a peak in the superficial layer that decreased in deeper layers. The direction of change was the opposite, with young rats demonstrating higher retardance in the superficial layer of the anterior segment, suggesting age‐dependent fibrillar density and alignment (*p* < 0.05) (Figures S2B, S4A and S8A).

### 3.2 Oestrogen

Oestrogen status affected the microarchitecture of the MCC. In the anterior segment's superficial layer, the oestrogen level was associated with retardance, and oestrogen deficiency (OVX) was associated with an increase in retardance in the pellet group of young rats (Figures S4A, S6A).

### 3.3 Dietary Loading

Significant differences in fibril orientation and retardation were observed due to diet. In the anterior segment's deep layer, significant differences in fibril orientation angles were evident between ovariectomised and non‐ovariectomised rats as the broad diet led to an increase in fibril orientation angles compared to rats on a powder diet (*p* < 0.05) (Figure S5A). Significant differences were observed in the non‐OVX old group between the pellet and powder diets, with the pellet diet leading to increased fibre orientation angles in the most superficial segments, particularly in layers 2 and 3 (Figure S5C).

#### 3.3.1 Fibril Orientation and Retardation

In the anterior and posterior segments, the older rats generally exhibited more variability and higher fibril orientation angles (*p* < 0.05) (Figures S5A, S7A,C). The anterior segment's superficial layer was significantly different between OVX young and old rats (Figure S7A), whereas in the deepest layer, a similar fibril orientation angle was observed in all age groups (Figure S5A), suggesting a dietary influence on collagen alignment.

A significant age‐related difference in fibril orientation angle was observed in the superficial layer of the posterior segment within the OVX groups, with the older rats showing significantly higher angles (*p* < 0.05) (Figure S5C). For the non‐OVX young and old rats in the board diet group, as well as all OVX young groups, the fibril orientation angle was consistently similar, suggesting an age‐transcending dietary effect.

Retardation within the anterior segment was coherent across groups (Figures S2B, S8A), but the most superior and posterior segments exhibited more variation between all groups (Figure S2D,F), with the middle layer of the most superior segment showing the most coherence in the board group (Figure S6B). In the most superior segment, retardation values in older non‐OVX rats on a pellet diet were significantly lower than those on a powder diet in layers 1 and 2 (*p* < 0.05, Figure S6B). Older rats showed significantly higher values of retardation in all sectors of layer 3, but lower values in layer 1 of the anterior segment, compared to the younger group. OVX rats showed increased retardation values in the anterior segment in layer 1 when compared to non‐OVX rats (*p* < 0.05, Figure S4C). In the posterior segment, there was a significant difference in orientation angles in younger rats in layer 2 and in older rats in layer 3, with the pellet diet leading to larger orientation angles than the board diet, but no significant difference was found in retardation values (Figures S5C, S6C).

## 4 Discussion

The aim of this study was to examine the effects of ageing, oestrogen level, and altered dietary loading on the collagen fibril orientation angle and organisation in rat MCC. Ageing was found to increase collagen fibril orientation angles, indicating structural disorganisation, while oestrogen deficiency (OVX) led to similar increases, suggesting that oestrogen plays a protective role in maintaining collagen structure. Dietary loading also increased fibril orientation angles in the diet board group, underscoring the role of mechanical stress in altering cartilage organisation. The hard diet led to consistent fibril orientation angles in the deepest layer regardless of age or oestrogen level. This consistent similarity in fibril orientation angles in the diet board group suggests that the hard diet promotes a stable collagen structure in the anterior and posterior segments, regardless of age or oestrogen levels. The present results extend these observations by providing a more detailed understanding of how these factors influence the collagen network at different regions and depths of the MCC. These findings are consistent with Yu et al. [11] and Orajärvi et al. [10], who demonstrated that mechanical loading and oestrogen levels play a critical role in regulating cartilage remodelling and structural integrity. The observed changes in collagen orientation and fibrocartilage thickness due to mechanical loading also align with the findings of Pirttiniemi et al. [13], who demonstrated that dietary loading significantly alters cartilage matrix composition through changes in Type I and Type II collagen distribution. The observed changes in collagen orientation and fibrocartilage thickness support previous findings, highlighting the role of mechanical and biochemical stimuli in cartilage adaptation and homeostasis [4, 13, 20, 23]. These findings also align with those of Teramoto et al. [12], who reported that compressive forces alter the extracellular matrix composition of the MCC, emphasising that mechanical stress is a critical regulator of cartilage organisation and function.

The anterior and posterior segments of the MCC displayed more variability in collagen fibril orientation angles, a finding that aligns with the work of Vanden Berg‐Foels et al. [21], who utilised helium ion microscopy for high‐resolution visualisation of the articular cartilage collagen network. This advanced imaging technique revealed detailed insights into the structural organisation of collagen, highlighting its variability in orientation depending on the depth and region of the tissue. The consistent fibril orientation angles in the most superior segment in this study suggest a more uniform mechanical environment or a different adaptation pattern in this area of the MCC. Vanden Berg‐Foels et al. [21] emphasised the importance of understanding collagen orientation for its role in resisting shear stresses and maintaining tissue integrity. Changes in collagen organisation can contribute to reduced tissue functionality and the development of joint diseases such as osteoarthritis [21].

Comparing the collagen architecture of the MCC to that of other joints is crucial, as the condylar cartilage exhibits unique properties. Unlike the hyaline cartilage of other synovial joints, the MCC is composed of fibrocartilage that confers distinct resilience and capacity for load distribution [1]. The observed differences in fibril orientation and retardation across the MCC depth may reflect the specialised functional adaptation of this fibrocartilage to the mechanical demands of the mandibular condyle, distinct from the hyaline cartilage typically studied in knee or hip joints.

As demonstrated by Hunziker et al. [19] and Rieppo et al. [20], collagen orientation is crucial for cartilage to withstand mechanical stresses and maintain joint integrity. Hunziker et al. [19], who used PLM to visualise the collagen network in knee joint cartilage, revealed how collagen orientation and density vary according to the mechanical environment, while Rieppo et al. [20] examined the stress‐relaxation response of patellar cartilage in relation to tissue composition and structure. Orajärvi et al. [10] observed that dietary consistency influences Type I and Type II collagen expression in condylar cartilage, supporting the conclusion that loading patterns critically influence cartilage adaptation. These findings align with those of Yu et al. [11], who demonstrated that reduced oestrogen levels in combination with altered mechanical loading exacerbate cartilage degradation, highlighting the importance of targeted interventions. The clinical implications of this study underscore the influence of mechanical and hormonal factors on the degenerative changes of TMJ [27].

The MCC is subject to a complex loading environment that varies significantly across its different anatomical segments. The anterior segment of the MCC is anatomically positioned to experience less mechanical loading compared to the most superior and posterior segments. The anterior segment likely deals with more shear forces due to its positioning and orientation relative to the direction of mandibular movement, whereas the superior and posterior segments encounter more direct compressive forces due to their load‐bearing function during occlusion and mastication. In the superficial layer of the anterior segment, oestrogen deficiency (OVX) led to a significant decrease in retardation in young rats, suggesting its critical role in maintaining the structural integrity of the MCC. Furthermore, as Kuroda et al. [2] elucidated, the biomechanical properties of the MCC are tailored to its functional requirements, with the different collagen types and their orientation reflecting adaptation to mechanical demands. In this context, the lower mechanical loading of the anterior parts may result in a different collagen composition or organisation, as suggested by the variation in fibril orientation angles and retardance profiles observed in the present findings. This is consistent with the work of Hunziker et al. [19] and Rieppo et al. [20].

A diet board maintained higher retardation across MCC segments, potentially influencing the onset of osteoarthritis through varied wear in the anterior segment compared to the most superior and posterior segments of the MCC. These mechanical loads influence the properties of cartilage and its ability to recover from stress, highlighting the importance of understanding these factors in developing targeted treatments for TMJ disorders. The research limitations encountered due to overlapping joints in the histological sections in some ROIs underscore the need for precise sampling and analysis to ensure accurate interpretations. The influence of developmental stages in young rats on the detection of significant dietary effects also highlights the importance of considering age‐related physiological changes in cartilage assessment.

The complicated architecture of the MCC plays a fundamental role in the biomechanics of the TMJ, particularly under the dynamic conditions imposed by ageing, hormonal changes, and mechanical loading. This study provides valuable insights into how these factors influence the structural integrity of the MCC, revealing significant associations that may contribute to our understanding of joint pathologies, such as osteoarthritis, advancing our understanding of how cartilage responds to physiological and mechanical factors. The present results underscore the profound influence of age, oestrogen, and dietary loading on the structural dynamics of rat MCC. The variation in retardation highlights the complex interplay of these factors, where ageing and oestrogen deficiency generally reduce collagen organisation, while diet hardness can either mitigate or exacerbate these effects.

## 5 Conclusions

Age emerged as the most significant factor influencing collagen fibril orientation, with older rats showing increased orientation angles, indicating age‐related structural changes in all layers of the MCC. Oestrogen levels also affected collagen architecture, with the older OVX old rats on the diet board showing higher fibril orientation angles. Dietary variations influenced collagen structure, especially in the deepest layer, with harder diets linked to increased fibril orientation angles and lower retardance values in the superior segment.

## Author Contributions

**Riikka Hauru:** analysing the data, conducting the experiment, writing. **Bijay Shakya:** analysing the data, writing. **Lassi Rieppo:** conducting the experiment, analysing the data, writing. **Anna‐Sofia Silvola:** conducting the experiment. **Jia Yu:** conducting the experiment. **Sakari Laaksonen:** conducting the experiment, writing. **Simo Saarakkala:** designing the study. **Aune Raustia:** designing the study, writing. **Pertti Pirttiniemi:** designing the study, writing.

## Ethics Statement

Animal experiments were approved by the National Project Authorisation Board of Finland. Permission number: ESAVI/7134/04.10.07/2015.

## Conflicts of Interest

The authors declare no conflicts of interest.

## Supporting information


**Appendix S1:** ocr70010‐sup‐0001‐AppendixS1.docx.

## Data Availability

The data that support the findings of this study are available from the corresponding author upon reasonable request.
