# Supplementary material for: Effects of Ageing, Oestrogen Level and Altered Dietary Loading on Rat Mandibular Cartilage—A Polarised Light Microscopy Study
Source: Orthod Craniofac Res. 2025 Aug 9;28(6):971–6. doi: 10.1111/ocr.70010 (PMC12603676; doi:10.1111/ocr.70010)
Supplement: Supplementary file 1 — Appendix S1: ocr70010‐sup‐0001‐AppendixS1.docx. [file OCR-28-971-s001.docx]

|  | | | |  |
| --- | --- | --- | --- | --- |
|  |  |  |  |  |
| **Age** | **Oestrogen** |  | **Dietary loading** | |
|  |  | Powder | Pellet | Diet board |
| **5-month-old** | OVX | 8 | 8 | 8 |
|  | non-OVX | 8 | 8 | 8 |
| **14-month-old** | OVX | 8 | 8 | 8 |
|  | non-OVX | 6 | 8 | 7 |

Appendix

Table S1. Distribution of Experimental Animals.

Figure S2

Figure S3. Boxplot of fibril orientation in different locations of the MCC according to three factors (age, oestrogen level and diet) *p<0.05.

**
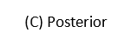

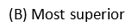

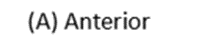

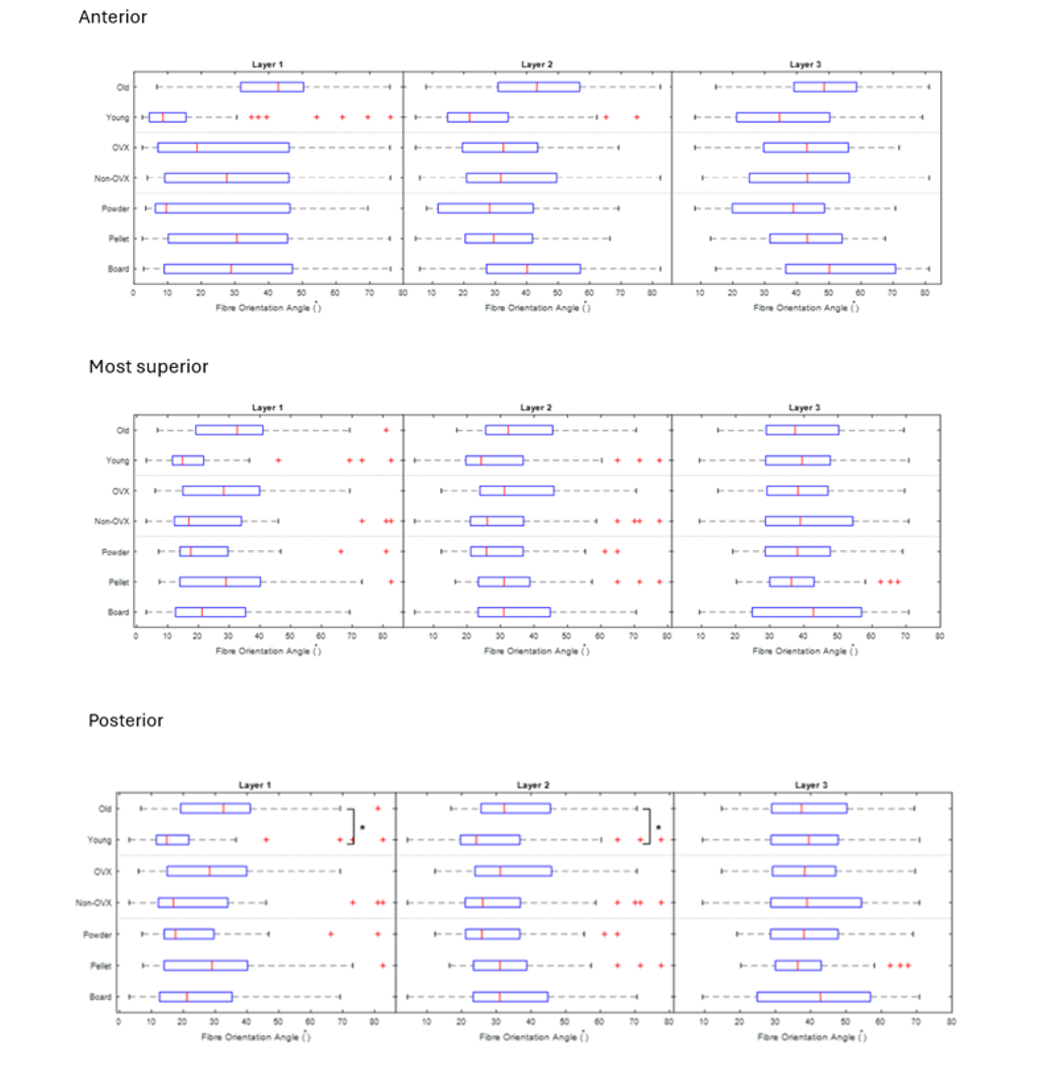
**

Figure S4. Boxplot of fibril retardation in different locations of the MCC according to three factors (age, oestrogen level and diet) *p<0.05.

**
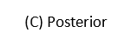

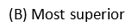

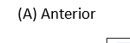

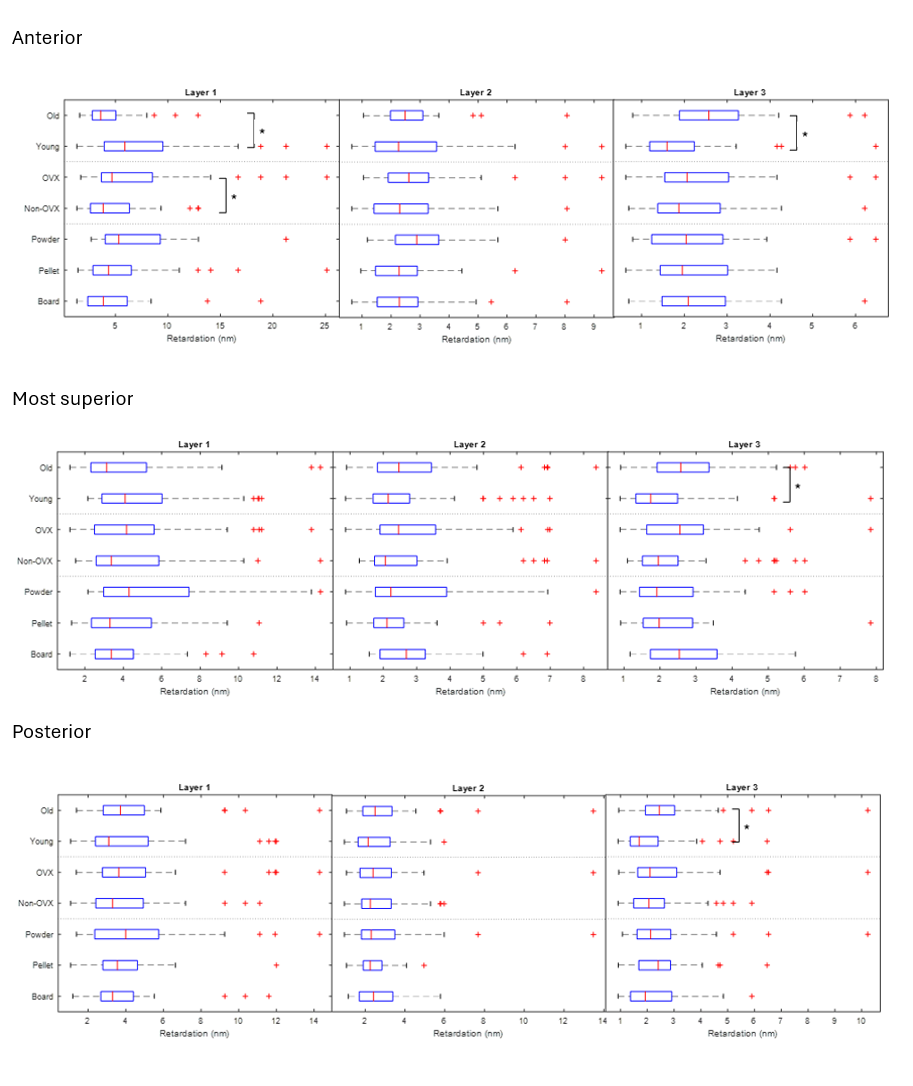
**

Figure S5. Boxplot showing fibril orientation angle distribution in different locations of the MCC when subgroups analyzed separately, *p<0.05.


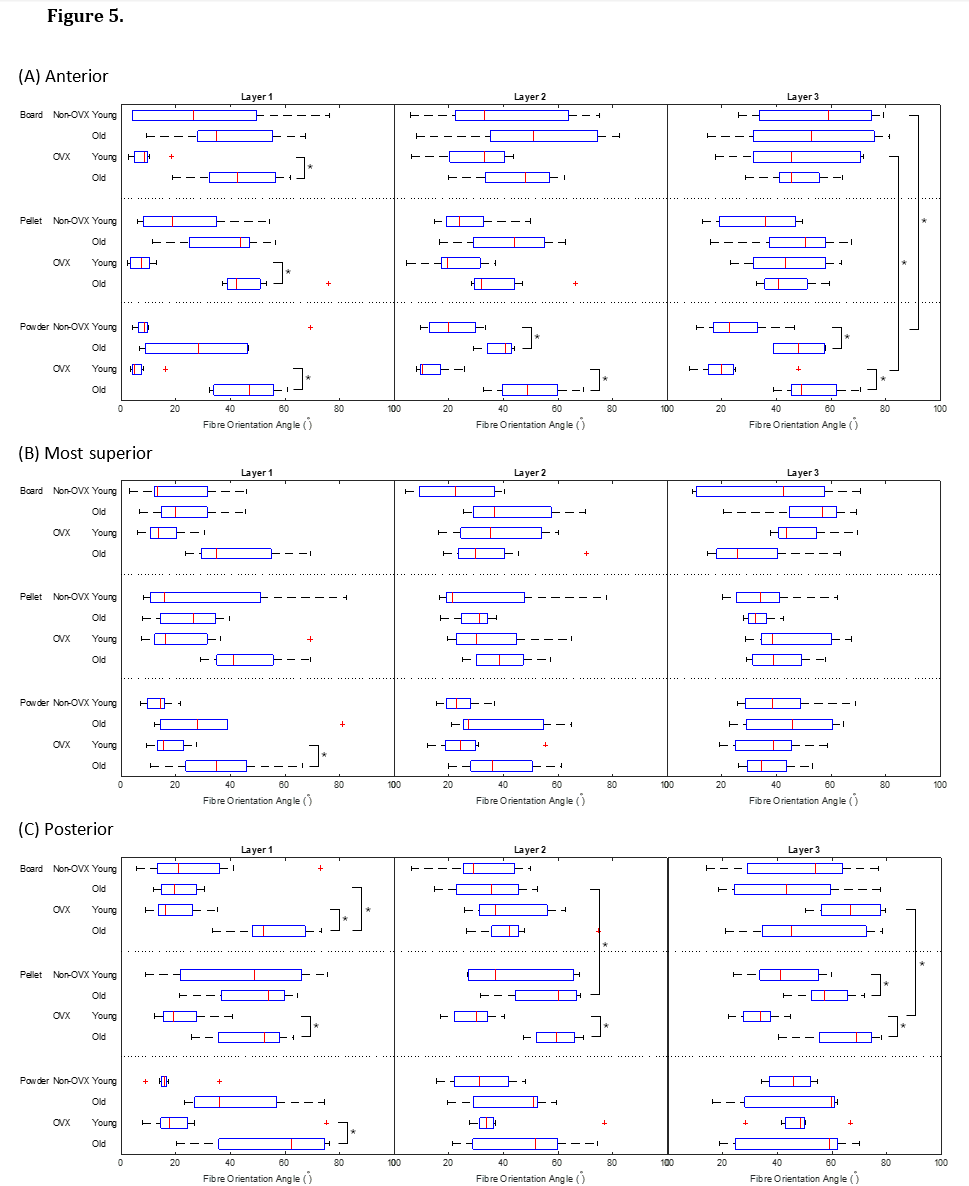


Figure S6. Boxplot showing fibril retardation distribution in different locations of the MCC when subgroups analyzed separately, *p<0.05.


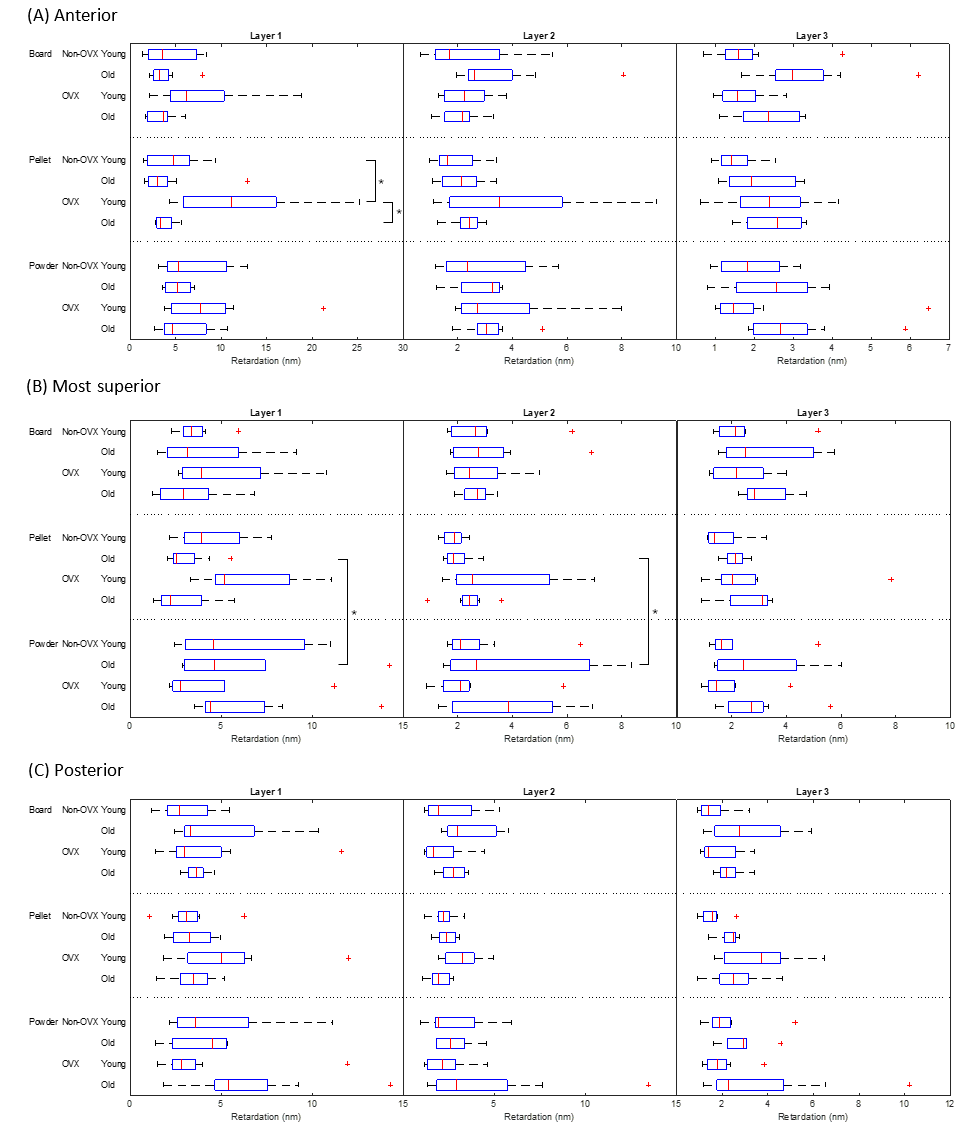


Figure S7. Significant differences of fibril orientation according to depth of MCC shown as p values, according to three factors (age, oestrogen level and diet).


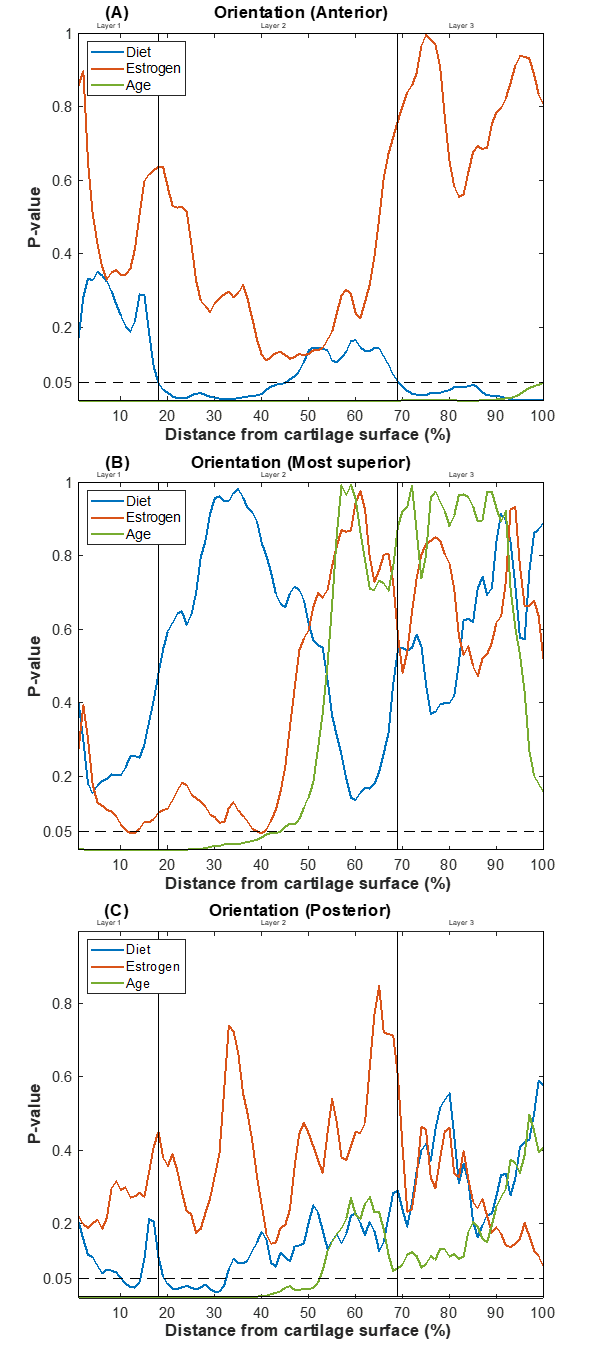


Figure S8. Significant differences of fibril retardation according to depth of MCC shown as p values, according to three factors (age, oestrogen level and diet).


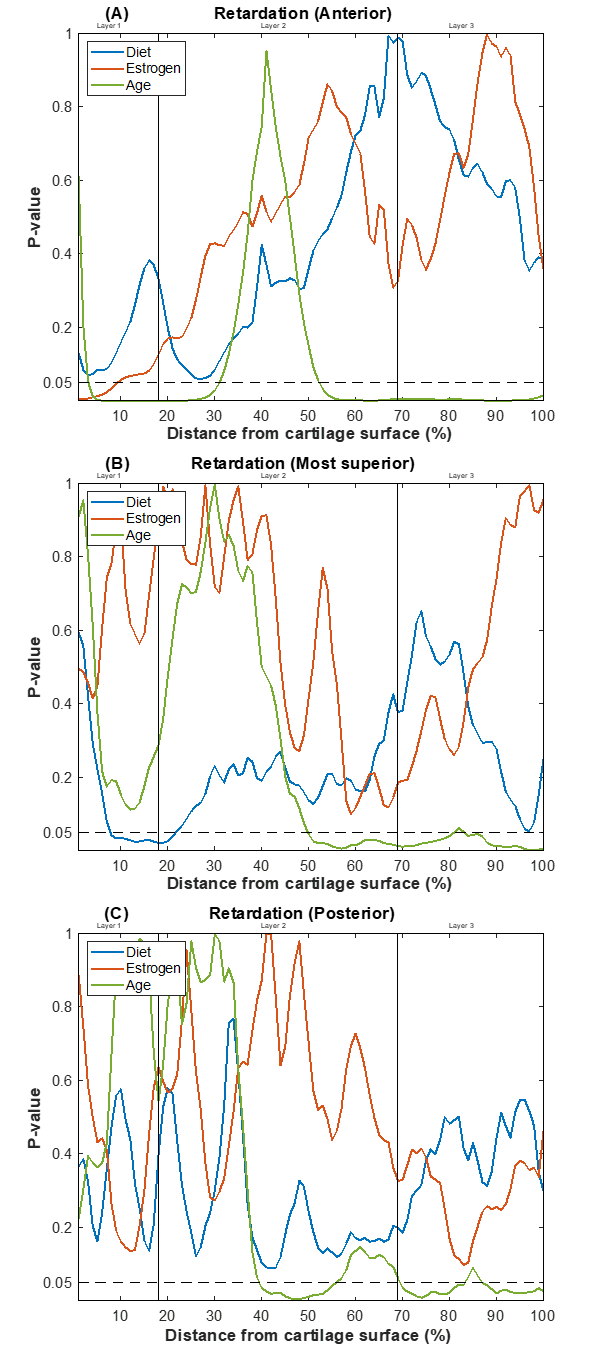


Figure S9. PLM image of a ovariectomized diet board group young rat MCC at 4× magnification.


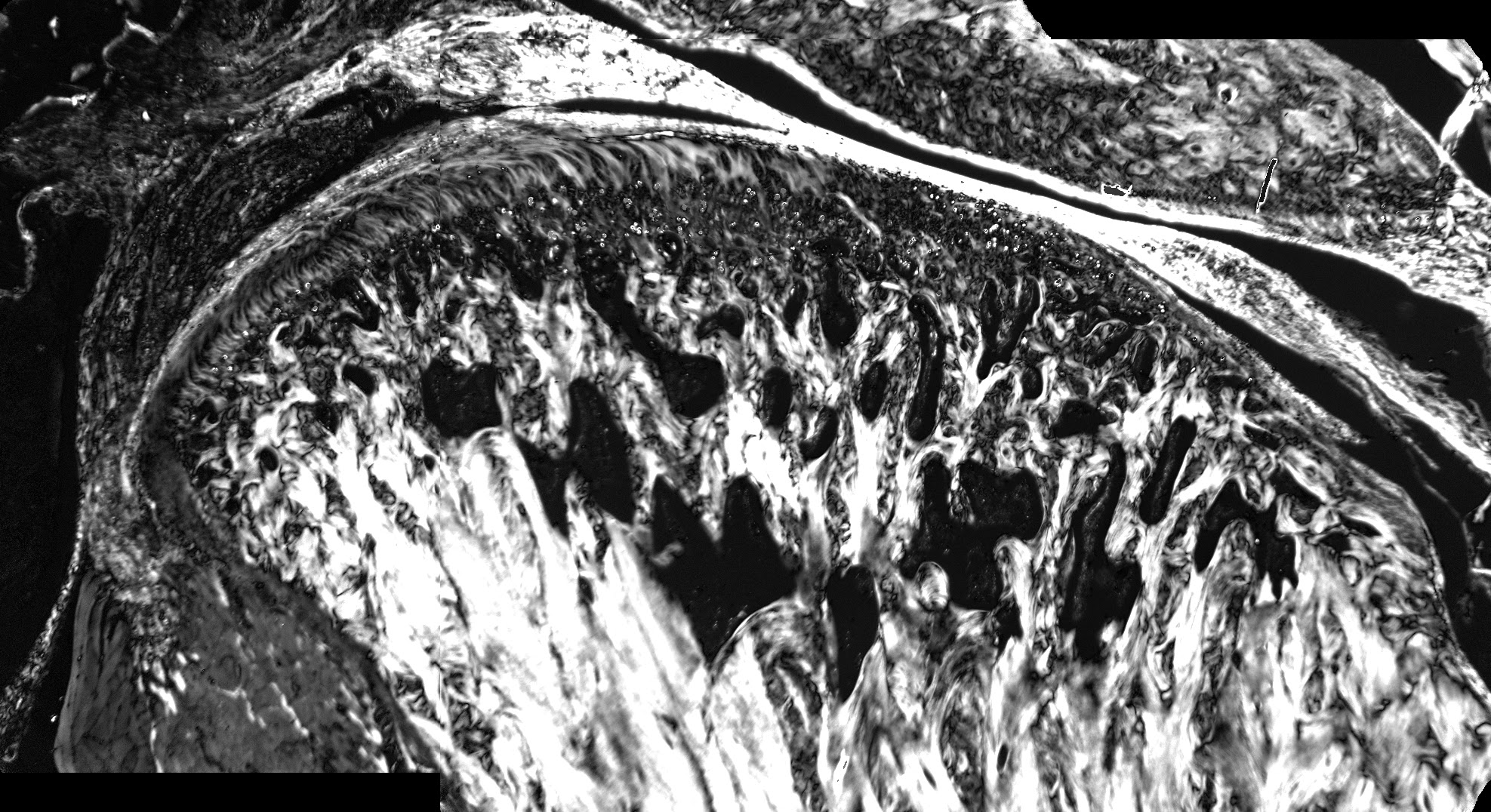


Figure S10. PLM images of young rats MCC at 4× magnification. A) ovx, diet board group, B) non-ovx, diet board group, C) ovx, pellet diet group, D) non-ovx, pellet diet group, E) ovx, soft diet group F) non-ovx, soft diet group.
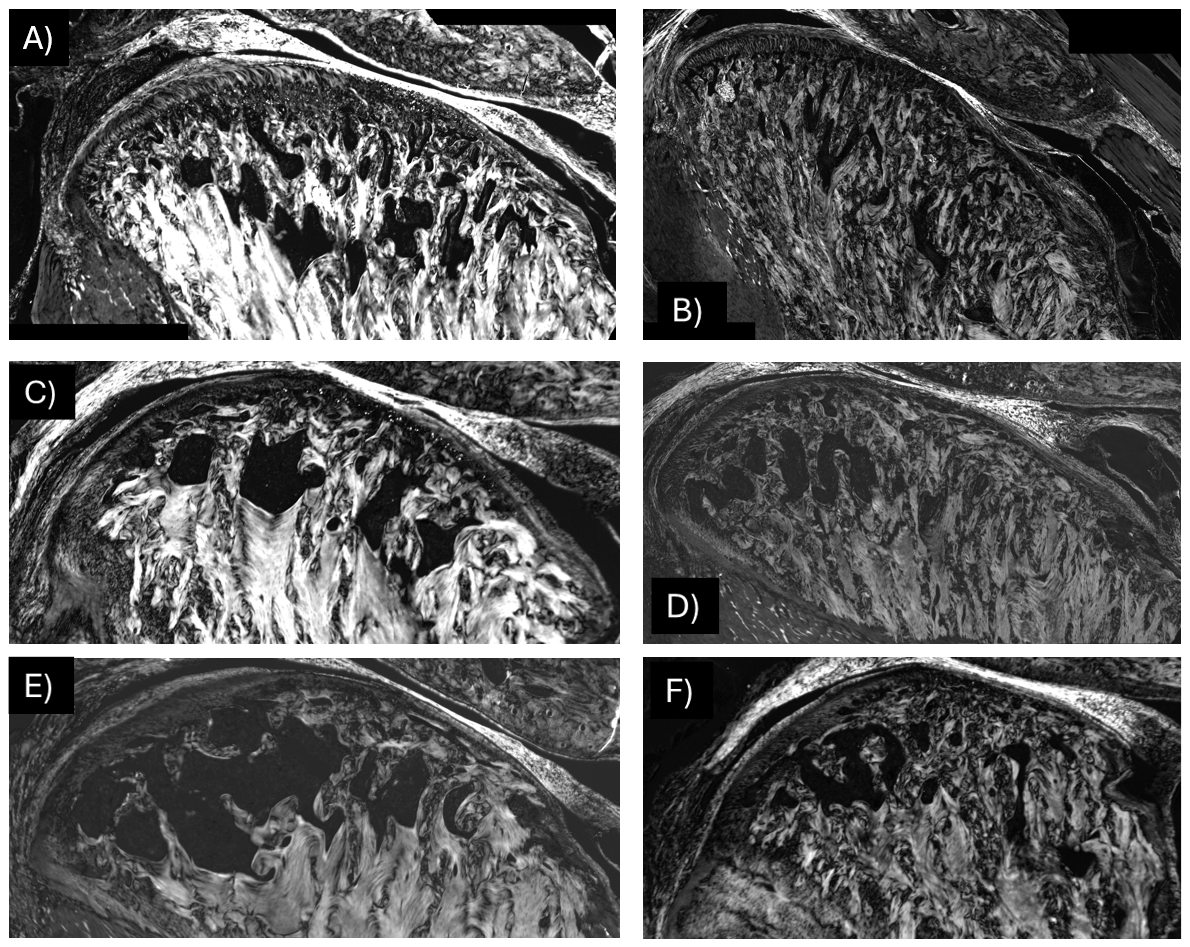


Figure S11. PLM images of old rats MCC at 4× magnification. A) ovx, diet board group, B) non-ovx, diet board group, C) ovx, pellet diet group, D) non-ovx, pellet diet group, E) ovx, soft diet group, F) non-ovx, soft diet group.


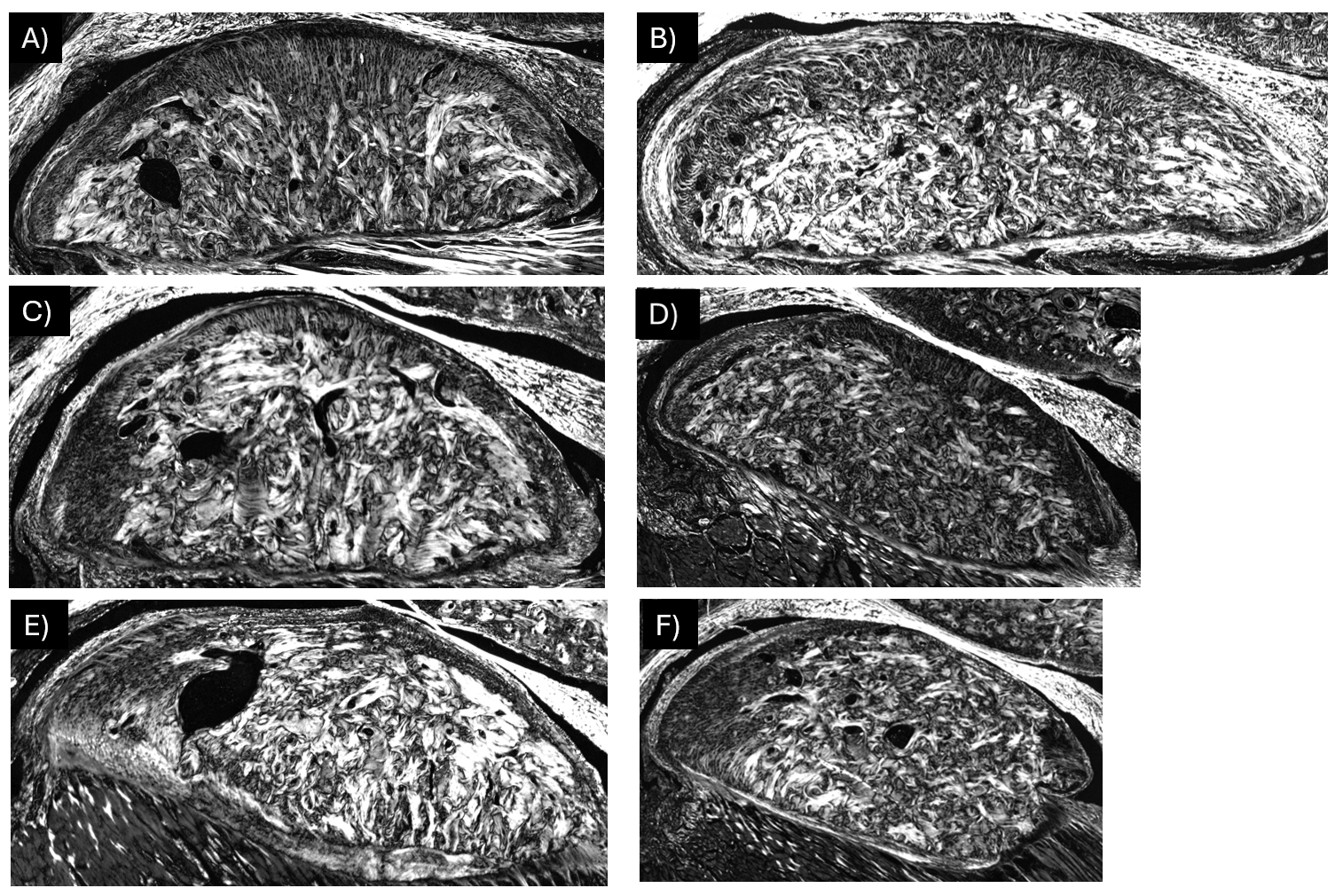


Figure S12. Hematoxylin and eosin staining and Pictosirius red staining images of old, non-ovx, pellet diet group rat MCC. A) Hematoxylin and eosin staining X 4 magnification. B) Pictosirius red staining X 4 magnification. C) Hematoxylin and eosin X 10 magnification. D) Pictosirius red staining X 10 magnification.


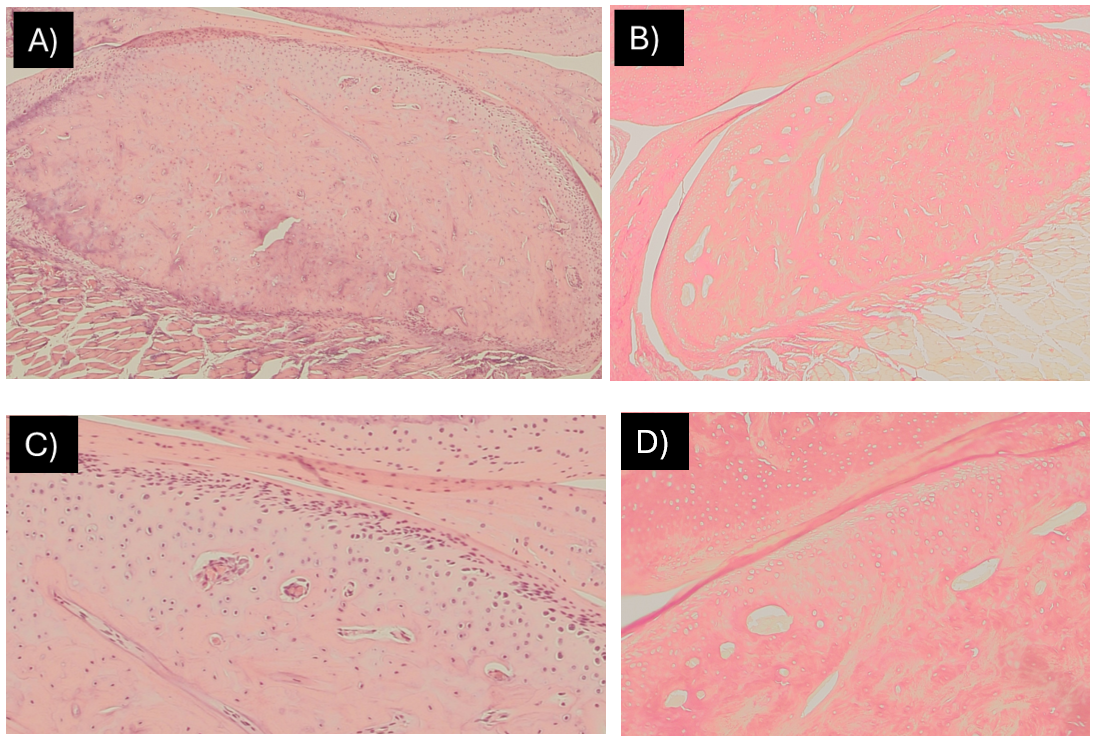


Figure S13. An example of a MATLAB GUI showing ROI selection for posterior segment on the left. The cropped image of the selected region is shown on the right along with the corresponding average orientation and retardation profiles.


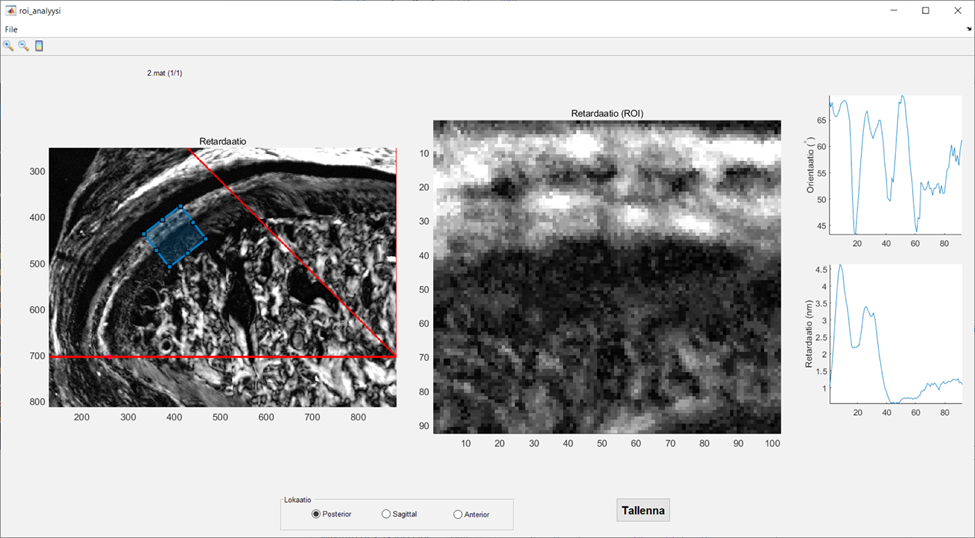


The orientation and retardation values were computed using a combination of polarized light microscopy (PLM) with the LC-PolScope technique and post-processing in MATLAB for visualization and analysis. The LC-PolScope system captures five intensity images of the sample under different polarization states: one with right circularly polarized light and four with elliptically polarized light whose principal axes are rotated in 45° increments. These images are processed using the inbuilt software that uses a mathematical algorithm to compute the orientation and retardance values. A detailed description of the algorithm is provided in a study by Mehta et al. (https://pmc.ncbi.nlm.nih.gov/articles/PMC3834771/). A background correction is applied to remove system-induced anisotropies by acquiring and processing a set of five reference images from a non-birefringent region of the sample.

Once the orientation and retardation values were computed, they are loaded into a custom-made MATLAB GUI along with the PLM image, enabling interactive region-of-interest (ROI) selection as shown in figure 1. To analyze specific regions of interest, a user-defined rectangular selection is applied, and subsequently rotated to align with the principal axis of the segment (anterior, most superior, posterior) under investigation. The orientation within the selected region is determined by computing the mean angular value across the width of the ROI at each vertical position which represents the orientation angle at each pixel within the selected region, and retardation is computed as the mean retardation value within the same ROI.
